# Antioxidant and anti-inflammatory properties of ginsenoside Rg1 for hyperglycemia in type 2 diabetes mellitus: systematic reviews and meta-analyses of animal studies

**DOI:** 10.3389/fphar.2023.1179705

**Published:** 2023-09-08

**Authors:** Qian Xie, Xiaoran Zhang, Qian Zhou, Yumei Xu, Lisha Sun, Qing Wen, Wei Wang, Qiu Chen

**Affiliations:** ^1^ Hospital of Chengdu University of Traditional Chinese Medicine, Chengdu, China; ^2^ School of Clinical Medicine, Chengdu University of Traditional Chinese Medicine, Chengdu, China; ^3^ School of Biomedical Sciences, Mianyang Normal University, Mianyang, China

**Keywords:** antioxidant and anti-inflammatory properties, ginsenoside Rg1, hyperglycemia, type 2 diabetes mellitus, systematic reviews and meta-analyses, animal studies

## Abstract

**Background:** According to existing laboratory data, ginsenoside Rg1 may help cure diabetes and its complications by reducing oxidative stress (OS) and managing inflammation. However, this conclusion lacks reliability and is unclear. As a result, the purpose of this systematic review and meta-analysis was to evaluate the antioxidant and anti-inflammatory effects of ginsenoside Rg1 in the treatment of diabetes and its complications.

**Methods:** We searched for relevant studies published through December 2022, including electronic bibliographic databases such as PubMed, EMBASE, Web of Science, CNKI, and Wanfang. The SYstematic Review Center for Laboratory Animal Experimentation Risk of Bias (SYRCLE RoB) tool was used to conduct a meta-analysis to assess the methodological quality of animal research. The meta-analysis was conducted using RevMan5.4 software, following the Cochrane Handbook for Systematic Reviews of Interventions. This study is registered in the International Systems Review Prospective Registry (PROSPERO) as CRD42023386830.

**Results:** Eighteen eligible studies involving 401 animals were included. Ginsenoside Rg1 was significantly correlated with blood glucose (BG), insulin levels, body weight, superoxide dismutase (SOD), malondialdehyde (MDA), tumor necrosis factor-α (TNF-α), and interleukin-6 (IL-6) levels. In addition, according to subgroup analysis, the hypoglycemic, anti-inflammatory, and antioxidant effects of ginsenoside Rg1 in type 2 diabetic animals were not affected by experimental species, modeling, experimental drug dosage, or course of treatment.

**Conclusion:** This meta-analysis presents a summary of the hypoglycemic effects of ginsenoside Rg1, which are achieved through anti-inflammatory and antioxidant mechanisms. These findings provide evidence-based support for the medical efficacy of ginsenoside Rg1. Specifically, ginsenoside Rg1 reduced MDA levels and restored SOD activity to exert its antioxidant activity. It had a positive effect on the reduction of IL-6 and TNF-α levels. However, the inclusion of studies with low methodological quality and the presence of publication bias may undermine the validity of the results. Further investigation with a more rigorous experimental design and comprehensive studies is necessary to fully understand the specific glycemic mechanisms of ginsenosides.

**Systematic Review Registration:** https://www.crd.york.ac.uk/PROSPERO/, identifier https://CRD42023386830.

## 1 Introduction

Ginseng, a perennial herb of the Acanthopanaceae family, has been documented in the ancient Shennong Herbal Classic for over 5,000 years (Baeg and So, 2013). A variety of diseases are treated with it in China due to its use in treating qi deficiency (Park et al., 2012). The main bioactive component of ginseng is ginsenosides (Yun, 2001). Ginsenosides are classified into three groups based on their chemical structures: protodiol (PPD), including Rb1, Rb2, Rb3, Rc, Rd, and Rg3; protriols (PPT), including Rg1, Re, Rf, and Rg2; and ginsenoside Ro (Song et al., 2017; Kim et al., 2018). A significant component of ginseng, ginsenoside Rg1, is relatively safe and has a low level of toxicity (Xie et al., 2015). Laboratory studies have shown that ginsenoside Rg1 has systemic effects and is therapeutic for a wide range of diseases. Ginsenoside Rg1 produces antioxidant and hepatoprotective effects by inducing the Keap1-Nrf2-ARE signaling pathway (Gao et al., 2017). Rg1 significantly attenuated multiple inflammatory responses in dextran sodium sulfate (DSS)-induced colitis in mice (Zhu et al., 2017). Meanwhile, ginsenoside Rg1 can treat H_2_O_2_-induced lens clouding through an anti-oxidative stress (OS) mechanism (Zhang et al., 2021). It acts in neurological disorders through multiple signaling pathways and related molecular mechanisms (Sun et al., 2022). In addition, rg1 reduces plasma cholesterol and triglyceride levels and inhibits the formation of aortic atherosclerosis, resulting in important benefits for cardiovascular disease (Xue et al., 2021). This article focuses on the role of ginsenoside Rg1 in T2DM. Rg1 restores glucose homeostasis and insulin sensitivity and attenuates obesity and insulin resistance. Rg1 further inhibits hepatic gluconeogenesis by preserving glucagon-impaired Akt activation while promoting Akt binding to FoxO1 and inactivating FoxO1 by phosphorylation (Liu et al., 2017). In addition, ginsenoside rg1 improved STZ-mediated diabetes in animals by reducing inflammatory cytokines (Alolga et al., 2020). At the same time, modern pharmacological studies have shown that ginsenoside rg1 has neuroprotective effects and can effectively alleviate diabetic peripheral neuropathy (Sun et al., 2022). These findings suggest that ginsenoside rg1 has beneficial effects on diabetes in terms of its antioxidant and anti-inflammatory properties.

Diabetes mellitus has become a worldwide public health problem in the 21st century due to the dramatic increase in the number of patients (Kitada et al., 2019). Globally, such diseases have spread due to population explosions, aging, urbanization, overweight, and lifestyle choices (Fernandez-Twinn et al., 2019). The main manifestation of diabetes is the development of long-term chronic hyperglycemia (Aryangat and Gerich, 2010). Studies have shown that chronic hyperglycemia greatly increases the risk of microvascular and macrovascular complications in diabetes, as well as mortality from cardiovascular disease (Nowaczewska et al., 2019). Glycated hemoglobin is a useful indicator of long-term blood sugar in the body. Therefore, actively controlling hyperglycemia and keeping glycated hemoglobin within the normal range is an important therapeutic tool for the effective treatment of diabetes and the prevention of its complications. The cause of type 2 diabetes mellitus is closely related to inflammation because bad diet and living habits lead to the accumulation of fat cells, causing a series of inflammatory reactions in the body, resulting in insulin resistance (Zozulinska and Wierusz-Wysocka, 2006; Alolga et al., 2020). According to the literature data, OS and inflammation may cause direct harm to the blood vessels of streptozotocin-induced diabetic animals, which could result in various complications (Zhao et al., 2014; Wronka et al., 2022). Most of the current drugs for the management of type 2 diabetes achieve the hypoglycemic effect by enhancing insulin sensitivity, promoting insulin secretion, inhibiting the reabsorption of terminal circulation, and supplementing exogenous insulin. Although blood glucose (BG) can be effectively controlled, the risk of hypoglycemia is high, and its complications remain inevitable. An increasing number of studies have shown that many hypoglycemic agents have antioxidant and anti-inflammatory properties. For example, metformin achieves its antioxidant and anti-inflammatory effects through the mechanism of AMPK activation (Dehkordi et al., 2019). In addition, we found that thiazolidinediones, sulfonylureas, α-glucosidase inhibitors, and glucagon-like peptide-1 receptor agonists have varying degrees of anti-inflammatory potential (Mathews et al., 2016). The study of traditional plant herbal extracts revealed that extracts, such as berberine and quercetin, have hypoglycemic, antioxidant, and anti-inflammatory properties (Li et al., 2015; Azeem et al., 2023). Therefore, the development of new drugs from antioxidant and anti-inflammatory mechanisms can effectively lower glucose hyperglycemia and prevent adverse events and complications.

There is currently no meta-analysis based on preclinical studies to summarize favorable evidence for ginsenoside rg1 in the treatment of type 2 diabetes. Furthermore, the results from animal trials are frequently influenced by several factors, which include small modeling sample numbers, modeling methodologies (Skovso, 2014), and intervention time. An umbrella review is a statistical analysis based on the review, analysis, sorting, and synthesis of the original literature, which is a method of comprehensive analysis of the results of previous similar studies. It integrates the results of previous studies in a standardized and quantitative manner, making evidence-based medical conclusions more reliable. Hence, we conducted a scientific review of these trial data to assess the antioxidant and anti-inflammatory potential of ginsenoside rg1 in the therapy of type 2 diabetes, which will help bridge the gap between animal research and clinical application and provide evidence support for future clinical work.

## 2 Materials and methods

This systematic review was conceived and is presented in the following paragraphs, the Preferred Reporting Items for Systematic Reviews and Meta-Analyses (PRISMA) (Page et al., 2021). The protocol concerning this study is obtainable at PROSPERO, CRD42023386830.

### 2.1 Search strategies

The electronic bibliographic information databases, comprising PubMed, EMBASE, Web of Science, Zhiwang, and Wanfang, were used for pertinent research published through December 2022. There are no language restrictions on retrieval. The medical subject terms (MeSH) and free terms used for database searches are [(“ diabetes” or “type 2 diabetes”) and (“ ginsenoside” or “ginsenoside rg1”) and (“animal” or “animal model” or “rat” or “mouse”)].

### 2.2 Inclusion criteria

1) Model: for all animal models with diabetes, blood sugar (BG) ≥ 11.1 mmol/L was considered as the criteria for successful modeling; 2) intervention: ginsenoside rg1 was given at any dose and duration; 3) control: the control group was given equal dose volume of non-functional sterile liquid (e.g., water and normal saline) or no treatment; 4) outcome: BG, insulin level, OS index, and inflammatory cytokines were observed; and 5) study design: it encompassed control study and separate control group.

### 2.3 Exclusion criteria

The exclusion criteria were as follows: 1) clinical trials or *in vivo* experiments; 2) the treatment group not treated with ginsenoside rg1; 3) control: other ginsenoside rg1 preparations (some medicinal preparations or supplements containing ginsenoside rg1); 4) study design: case study, crossover study, and study without a separate control group; 5) not original or incomplete research papers; 6) repeated release; and 7) no full-text study available.

### 2.4 Research selection and data extraction

All searched articles are entered into EndNote X9, and duplicate articles are deleted. Two investigators independently conducted literature collection according to the inclusion and exclusion criteria. Initially, titles and abstracts were selected to preclude extraneous articles. After the initial screening, potentially eligible articles underwent full-text screening for final determination.

Two evaluators abstracted the following messages from the enrolled studies: 1) basic data: initial author’s surname, name, and year of publication; 2) features of the experimental animals, which included animal type, sex, sample size, age, and weight; 3) modeling approach and criteria for successful modeling; 4) treatment information: administration method, source, duration, and dose of intervention drugs; 5) and outcome indicators: BG, insulin levels, TNF-α, IL-6, MDA, and SOD. Analyses of indicators, such as IL-1β, ROS, and GSH, were abandoned due to the inadequacy of the included experiments. All resultant measures were continuous data; therefore, means and standard deviations were drawn for each intervening group. We built a database and hand-pulled data from the collected papers. If the results were presented only in the form of graphs, we attempted to contact the authors for more details. If there was no response, graphical data were quantified using WebPlotDigitizer4.5 software (https://automeris.io/WebPlotDigitizer).

If results were presented at more than one time point, data were retrieved from the final time point. If the drug involved more than one dose in the treatment group, we extracted only the data for the highest dose. At each stage, two evaluators independently assessed and extracted each study. Disagreements between the two investigators about whether the study should be integrated and the data extracted were addressed through discussions with a third evaluator.

### 2.5 Bias risk assessment

We evaluated the methodological quality of the enrolled research using the SYstematic Review Center for Laboratory Animal Experimentation Risk of Bias (SYRCLE RoB) tool (Hooijmans et al., 2014). The SYRCLE RoB tool for animal research comprises 10 programs based on six different types of biases. The maximum score for individual studies was 10 points. Any discrepancies that arose during the quality assessment process were ultimately resolved through negotiation with the appropriate authors.

### 2.6 Statistical analysis

The meta-analysis was performed using RevMan 5.4 software. All outcome indicators were continuous variables (e.g., BG, serum, and insulin levels). Therefore, the combined total effect sizes of the results were expressed using standardized mean differences (SMD) and 95% confidence intervals (CI), and *p*-values <0.05 were deemed statistically meaningful. Heterogeneity between studies was evaluated using I-squared (I^2^), with a fixed-effects model combining effect sizes for I^2^ ≤ 50%. I^2^ > 50% was considered to represent substantial heterogeneity, and a random-effects model was utilized to combine effect sizes. If sufficient studies were available, subgroup analyses were performed to identify sources of heterogeneity based on the following variables: species (rats, mice), STZ dose (≤50 and ≥50 mg/kg), drug dose (<40 mg/kg/day, ≥40 mg/kg/day), and intervention duration (<8 weeks, ≥8 weeks). A sensitivity analysis was conducted to evaluate whether separate studies would impact the total effect size by excluding one study at each stage to appraise the stability of the overall outcome. If there were at least 10 studies per outcome, potential publishing bias was evaluated using funnel plots and Egger tests (Egger et al., 1997). In addition, trimming and padding methods were conducted in the presence of publication bias.

## 3 Results

### 3.1 Research inclusion

**FIGURE 1:**
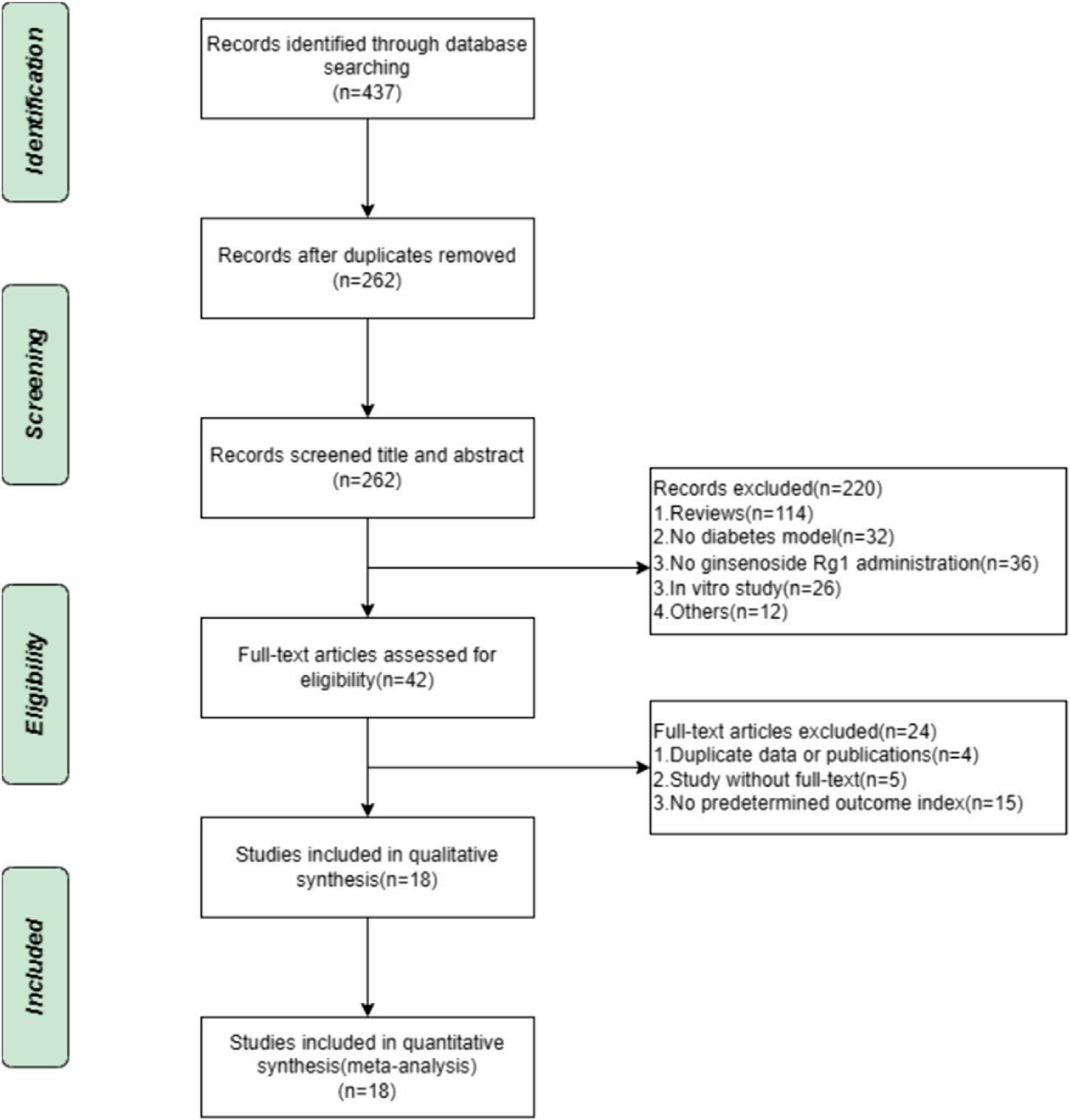
Flowchart for inclusion of studies.

A systematic evaluation and meta-analysis of the search database resulted in the establishment of 437 animal studies. After the elimination of duplicates, 262 publications were left. Of the titles and abstracts screened, 220 publications were rejected for the following factors: 1) review articles, 2) not diabetic animal model, 3) no interventions using ginsenoside rg1, 4) *in vitro* studies, and 5) others. The remaining 42 animal studies were then screened for full text. A total of 24 studies were found to be non-compliant for the following reasons: 1) duplicate data or publications (*n* = 4), 2) studies without full text (*n* = 5), and 3) the absence of predetermined outcome indicators (*n* = 15). Ultimately, 18 eligible projects were included in this systemic evaluation. The selection process is depicted in Figure 1.

### 3.2 Research characteristics

Eighteen studies published between 2010 and 2022 were accepted: nine in English (Yang et al., 2012; Yu et al., 2016; Tian et al., 2017; Yin and Wang, 2017; Yuan et al., 2019; Gao et al., 2020; Liu et al., 2021; Ma et al., 2022; Peng et al., 2022) and nine in Chinese (Jie, 2010; Xin, 2015; Jianchao, 2016; Zheng Yongren, 2016; Chen Xianren, 2020; Yang Jing, 2020; Eshu, 2021; Wu Lina, 2021; Jie, 2022). A total of 401 animals were involved, of which the test group comprised 203 animals and the control group comprised 198 animals. The animal species consisted of rats and mice, with six studies (33.3%) using mice and 12 studies (66.7%) using rats. In all studies, body weights were 180–280 g for rats and 20–26 g for mice. Four studies did not report animal weights. Multiple levels of ginsenoside rg1 dosing ranging from 1 to 100 mg/kg/day were implemented in these studies, and two studies did not report ginsenoside rg1 dosing. The control group consisted mainly of the same solvent, such as saline. Intervention durations were <8 weeks and ≥8 weeks, with durations ranging from 4 to 12 weeks. The intervention duration was <8 weeks in five studies (27.8%) and ≥8 weeks in 11 studies (61.1%), and two studies did not report the intervention duration. Table 1 describes the basic features of the 18 studies. Furthermore, a summary table depicting ginsenoside rg1 is displayed in Supplementary Table S2.

**TABLE 1:** Characteristics of the included studies.

Study (year)	Species (sex, *n* = treatment/control group, age, and weight)	Modeling method and standard	Ginsenoside Rg1 intervention (administration, drug dose, and duration)	Outcomes
Feng Jie (2010)	ICR mice (male, 10/10, 22 ± 3 g)	Tail vein injection of alloxan (85 mg/kg) BG > 11.1 mmol/L	By gavage, 10/20/40 mg/kg/d, 4 weeks	1. BG
				2. Weight
				3. SOD
Zhen et al. (2016)	SD rats (male, 12/12, aged 8 weeks, 200 ± 20 g)	Intraperitoneal injection of STZ (55 mg/kg) + HSFD	By gavage, 20/40/80 mg/kg/d, 6 weeks	1. BG
				2. Insulin
Yao Jianchao (2016)	SD rats (male, 12/12)	HSFD FBG ≥ 7.8 mmol /L or RBG≥ 11.1 mmol /L	By gavage, 2/4/8 mg/kg/d, 8 weeks	1. BG
				2. TNF-α
				3. IL-6
				4. SOD
				5. MDA
Ruan et al. (2021)	SD rats (male, 6/6, 180∼200 g)	Intraperitoneal injection of STZ (35 mg/kg) + HSFD	By gavage, 25/50/100 mg/kg/d, 4 weeks	1. TNF-α
				2. IL-6
Yang Jing (2020)	SD rats (male, 20/20, 200 ± 20 g)	Intraperitoneal injection of STZ (55 mg/kg) BG > 16.7 mmol/L	By gavage, 21 mg/kg/d, 12 weeks	1. SOD
				2. MDA
Li et al. (2015)	SD rats (10/10, 200∼220 g)	Intraperitoneal injection of STZ (30 mg/kg) + HSFD FBG ≥ 7.8 mmol/L or RBG ≥ 11.1 mmol/L	By gavage, 2/4/8 mg/kg/d, 8 weeks	1. BG
				2. TNF-α
				3. IL-6
				4. SOD
				5. MDA
Wu Lina (2021)	SD rats (male, 10/10, 180∼200 g)	Intraperitoneal injection of STZ (40 mg/kg) + HSFD FBG ≥ 11.1 mmol/L	By gavage, 10/30 mg/kg/d, 8 weeks	1. TNF-α
				2. IL-6
Li et al. (2022)	C57BL/6J mice (male, 8/8, aged 8 weeks, 20∼26 g)	Intraperitoneal injection of STZ (110 mg/kg) + HFD BG > 16.7 mmol/L	By gavage, 1/5/10 mg/kg/d, 8 weeks	1. BG
Chen Xianren (2020)	SD rats (male, 15/15, aged 3 weeks, 160∼200 g)	Intraperitoneal injection of STZ (30 mg/kg) + HSFD BG ≥ 16.7 mmol/L	By gavage, 10 mg/kg/d, 8 weeks	1. BG
				2. SOD
				3. MDA
Ma et al. (2022)	C57BL/6J mice (10/10, aged 4 weeks)	Intraperitoneal injection of STZ (60 mg/kg) BG > 16.7 mmol/L	By gavage	1. TNF-α
				2. IL-6
Yang et al. (2012)	C57BL/6J mice (male, 23/23, aged 7 weeks, 20–25 g)	Intraperitoneal injection of STZ (50 mg/kg) FBG > 11.1 mmol/L	By gavage, 10 mg/kg/d, 4 weeks	1. BG
				2. IL-6
Gao et al. (2019)	40 db/db mice and 10 wild-type mice (male, 10/10, aged 22 weeks)	Spontaneous diabetic model	By gavage, 25/50 mg/kg/d, 8 weeks	1. BG
				2. SOD
Yuan et al. (2019)	6/6	Intraperitoneal injection of STZ (35 mg/kg) BG > 16.7 mmol/L	By gavage	1. BG
Liu et al. (2021)	SD rats (male, 8/8, aged 8 weeks, 180–200 g)	Intraperitoneal injection of STZ (50 mg/kg)	By intraperitoneal injection, 50 mg/kg/d, 8 weeks	1. TNF-α
				2. IL-6
				3. SOD
				4. MDA
Yaoyao Yin and Junxia Wang, 2017	SD rats (male, 10/10, 210–280 g)	Intraperitoneal injection of STZ (65 mg/kg) BG > 16.7 mmol/L	By gavage, 50 mg/kg/d, 8 weeks	1. BG
				2. TNF-α
Tian et al. (2017)	SD rats (male, 10/10, aged 4 weeks, 200 ± 20 g)	Intraperitoneal injection of STZ (30 mg/kg) + HFD BG > 16.7 mmol/L	By gavage, 25/50 mg/kg/d, 8 weeks	1. BG
				2. Insulin
				3. TNF-α
				4. IL-6
Yu et al. (2015)	Wistar rats (male, 15/10, aged 4 weeks, 200±20 g)	Intraperitoneal injection of STZ (40 mg/kg) + HSFD BG > 16.7 mmol/L	By intraperitoneal injection, 10/15/20 mg/kg/d, 12 weeks	1. BG
Peng et al. (2022)	SD rats (male, 8/8, adult, 180–200 g)	Intraperitoneal injection of STZ (35 mg/kg) + HFDB G > 16.7 mmol/L	By gavage, 25/100 mg/kg/d, 4 weeks	1. BG
				2. Insulin
				3. TNF-α
				4. IL-6
				5. SOD
				6. MDA

Abbreviations: BG, blood glucose; TNF-α, tumor necrosis factor-α; IL-6, interleukin-6; SOD, superoxide dismutase; MDA, malondialdehyde; STZ, streptozocin; Scr, serum creatinine; HFD, high-fat diet; HSFD, high-sugar and high-fat diet.

### 3.3 Research quality

We evaluated the quality of the included studies. The overall quality of each study was low, with a range of three–five points. Two studies obtained six points, three studies obtained five points, nine studies obtained four points, and four studies obtained three points. Of the 18 included studies, two studies (11.1%) mentioned random sequence generation, eight studies (44.4%) reported baseline characteristics in full, six studies (33.3%) reported allocation concealment, and five studies (27.8%) described randomized captivity. All studies did not report blinded allocation, randomization of outcome evaluation, or blinding of outcomes. All of these studies have comprehensive outcome-based data and published intended results. Concerning other sources of bias, all studies showed no conflict of interest between authors. Methodologically relevant qualities regarding the incorporated studies are summarized in Table 2.

**TABLE 2:** Risk of bias of included studies.

Study (year)	A	B	C	D	E	F	G	H	I	J	Total
Feng Jie, 2010	−	+	?	−	−	−	−	+	+	+	4
Zhen et al., 2016	−	−	?	+	−	−	−	+	+	+	4
Yao Jianchao, 2016	−	−	−	−	−	−	−	+	+	+	3
Ruan et al., 2021	−	+	−	−	−	−	−	+	+	+	4
Yang Jing (2020)	−	−	−	−	−	−	−	+	+	+	3
Li et al. (2015)	−	−	+	?	−	−	−	+	+	+	4
Wu Lina (2021)	−	−	?	+	−	−	−	+	+	+	4
Li et al., 2022	−	−	−	−	−	−	−	+	+	+	3
Chen Xianren (2020)	−	−	+	−	−	−	−	+	+	+	4
Ma et al. (2022)	−	−	−	?	−	−	−	+	+	+	3
Yang et al. (2012)	−	+	−	−	−	−	−	+	+	+	4
Gao et al., 2019	−	−	+	−	−	−	−	+	+	+	4
Yuan et al. (2019)	−	−	+	+	−	−	−	+	+	+	5
Liu et al. (2021)	+	+	?	?	−	−	−	+	+	+	5
Yaoyao Yin and Junxia Wang, 2017	−	+	−	−	−	−	−	+	+	+	4
Tian et al. (2017)	−	+	+	+	−	−	−	+	+	+	6
Yu et al., 2015	+	+	?	+	−	−	−	+	+	+	6
Peng et al. (2022)	−	+	+	?	−	−	−	+	+	+	5

A, sequence generation; B, baseline characteristics; C, allocation concealment; D, random housing; E, blinding of experimentalists; F, random for outcome assessment; G, blinding of outcome assessors; H, incomplete outcome data; I, selective outcome reporting; J, other biases; +, low risk of bias; −, high risk of bias; ?, unclear risk of bias.

### 3.4 Effect of ginsenoside Rg1 on blood glucose

**FIGURE 2:**
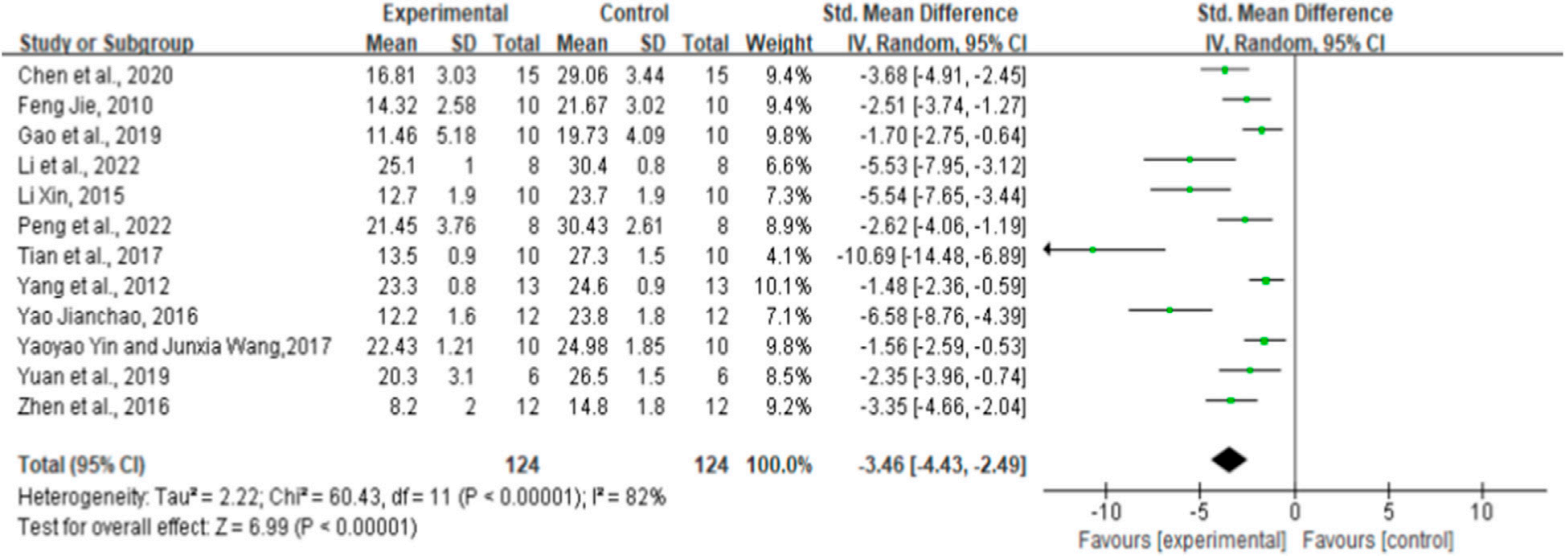
Forest plot: effect of ginsenoside Rg1 on blood glucose.

Twelve randomized controlled trials showed the effect of ginsenoside rg1 on BG. The aggregated results show that ginsenoside rg1 significantly reduced BG levels compared to controls [*n* = 248, SMD = −3.46, 95% CI (−4.43, −2.49), *p* < 0.00001; heterogeneity: X^2^ = 60.43, *p* < 0.00001; I^2^ = 82%, Figure 2]. Subgroup analysis was performed based on the type of animal model, intervention duration, drug dose, and STZ dose. Additional beneficial effects were noted when rats (*p* < 0.001), STZ doses <50 mg/kg (*p* < 0.001), intervention durations ≥8 weeks (*p* < 0.001), and ginsenoside rg1 doses <40 mg/kg (*p* < 0.001) were studied (Supplementary Table S3). For the BG subgroup, analyses did not reveal sources of heterogeneity between studies, and significant heterogeneity remained. In addition, visual inspection of funnel plots revealed asymmetric effects of ginsenoside rg1 on BG (Supplementary Figure S1), whereas the outcome of the Egger test was statistically significant [intercept: −7.90, 95% CI (−8.44, −4.73); *p* = 0.000] (Supplementary Figure S1).

### 3.5 Effect of ginseng Rg1 on insulin levels

**FIGURE 3:**
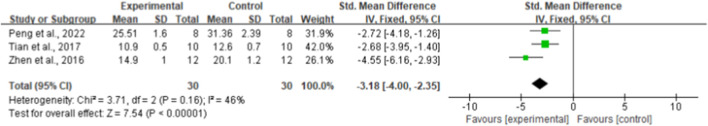
Forest plot: effect of ginseng Rg1 on insulin levels.

By combining the effect sizes of the three pairwise comparisons, a significant reduction in insulin levels was observed after ginseng rg1 administration compared with the control group [*n* = 60, SMD = −3.18, 95% CI (−4.00, −2.35), *p* < 0.00001; heterogeneity: X^2^ = 3.71, *p* = 0.16; I^2^ = 46%, Figure 3]. In addition, no publication bias was applied to insulin levels because fewer than 10 studies were included.

### 3.6 Effect of ginsenoside Rg1 on TNF-α

**FIGURE 4:**
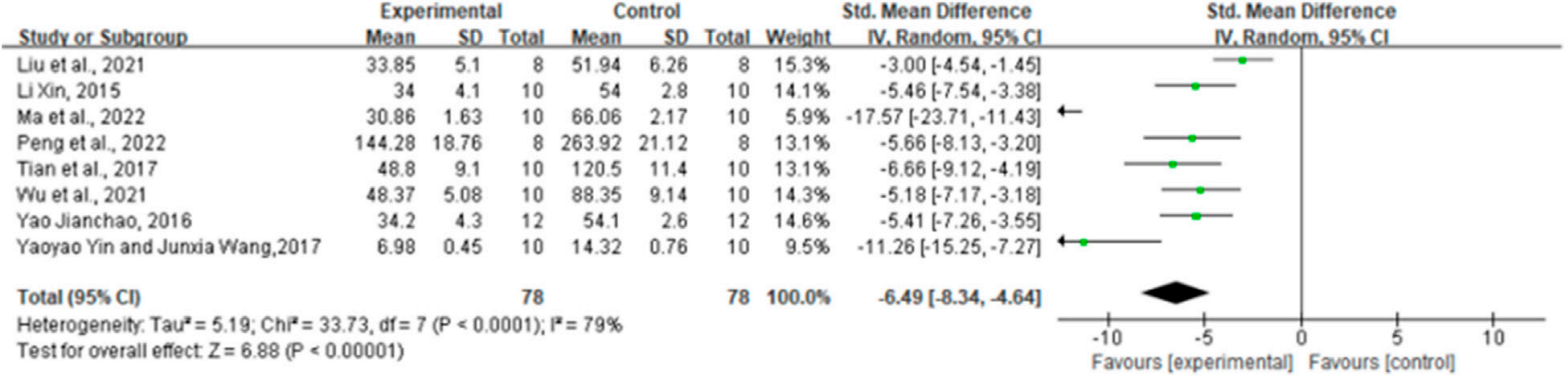
Forest plot: effect of ginseng Rg1 on TNF-α.

By combining the effect sizes of the eight pairwise comparisons, TNF-α levels were significantly reduced after ginsenoside rg1 administration compared to control [*n* = 156, SMD = −6.49, 95% CI (−8.34, 4.64), *p* < 0.00001; heterogeneity: X^2^ = 33.73, *p* < 0.0001; I^2^ = 79%, Figure 4]. Subgroup analysis was performed based on the animal model type, intervention duration, drug dose, and STZ dose. Additional beneficial effects were noted when mice (*p* < 0.001), STZ doses ≥50 mg/kg (*p* = 0.02), intervention durations ≥8 weeks (*p* < 0.001), and ginsenoside rg1 doses ≥40 mg/kg (*p* < 0.001) were studied (Supplementary Table S3). The results of the subgroup analysis indicate that drug and STZ doses may be the source of TNF-α heterogeneity. Moreover, no publication bias was applied to TNF-α because fewer than 10 studies were included.

### 3.7 Effect of ginsenoside Rg1 on IL-6

**FIGURE 5:**
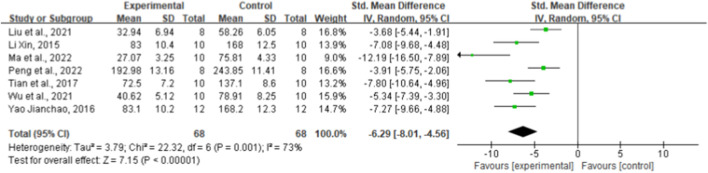
Forest plot: effect of ginseng Rg1 on IL-6.

Regarding the effect on IL-6, seven randomized controlled trials showed the effect of ginsenoside rg1 on this outcome. The aggregated results showed that ginsenoside rg1 significantly decreased IL-6 levels compared to controls [*n* = 136, SMD = −6.29, 95% CI (−8.01, 4.56), *p* < 0.00001; heterogeneity: X^2^ = 22.32, *p* = 0.001; I^2^ = 73%, Figure 5]. Subgroup analysis was performed based on animal model type, intervention duration, drug dose, and STZ dose. Additional beneficial effects were noted when mice (*p* < 0.001), STZ doses ≥50 mg/kg (*p* < 0.001), intervention durations ≥8 weeks (*p* < 0.001), and ginsenoside rg1 doses <40 mg/kg (*p* < 0.001) were studied (Supplementary Table S3). The results of the subgroup analysis indicate that drug dose may be a source of IL-6 heterogeneity. In addition, no publication bias was applied to IL-6 because fewer than 10 studies were included.

### 3.8 Effect of ginsenoside Rg1 on superoxide dismutase

**FIGURE 6:**
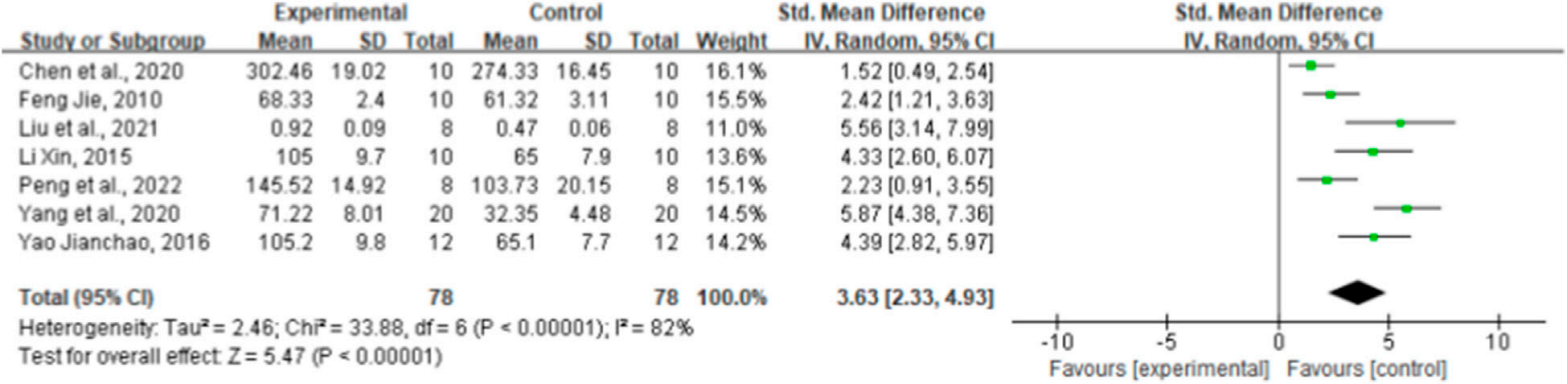
Forest plot: effect of ginseng Rg1 on SOD.

Seven paired comparisons mentioned the effect of ginsenoside rg1 on SOD. The aggregated results show that ginsenoside rg1 significantly increased SOD levels compared to the control [*n* = 156, SMD = 3.63, 95% CI (2.33, 4.93), *p* < 0.00001; heterogeneity: X^2^ = 33.88, *p* < 0.00001; I^2^ = 82%, Figure 6]. Subgroup analysis was performed based on animal model type, intervention duration, drug dose, and STZ dose. Studies showed more beneficial effects when using rats (*p* < 0.001), STZ doses ≥50 mg/kg (*p* < 0.001), intervention durations ≥8 weeks (*p* < 0.001), and ginsenoside rg1 doses <40 mg/kg (*p* < 0.001) (Supplementary Table S3). The results of the subgroup analysis indicate that the STZ dose and intervention duration may be a source of SOD heterogeneity. In addition, publication bias was not applied to SOD because fewer than 10 studies were included.

### 3.9 Effect of ginsenoside Rg1 on malondialdehyde

**FIGURE 7:**
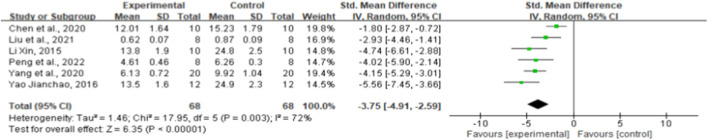
Forest plot: effect of ginseng Rg1 on MDA.

MDA effect sizes were calculated using data from six animal trials. Ginsenoside rg1 resulted in significantly lower MDA levels [*n* = 136, SMD = −3.75, 95% CI (−4.91, −2.59), *p* < 0.00001; heterogeneity: X^2^ = 17.95, *p* = 0.003; I^2^ = 72%, Figure 7]. The included studies were stratified based on several variables, such as intervention duration, drug dose, and STZ dose. More positive effects were observed when studies used STZ doses ≥50 mg/kg (*p* < 0.001), when intervention duration was <8 weeks (*p* < 0.001), and when ginsenoside rg1 doses <40 mg/kg were used (*p* < 0.001) (Supplementary Table S3). The findings of the subgroup analysis imply that the sources of MDA heterogeneity may be STZ and medication doses. In addition, publication bias was not applied to MDA because fewer than 10 studies were included.

### 3.10 Sensitivity analysis

Sensitivity analyses for BG, TNF-α, IL-1β, SOD, and MDA were performed separately by deleting one study at each stage and identifying that none of the studies significantly affected the combined effect size.

## 4 Discussion

### 4.1 Research purpose

The purpose of this study was to assess the active effect of ginsenoside rg1 on T2DM model animals and its underlying mechanisms. A total of 18 studies comprising 437 animals were involved, and the results from this systematic evaluation indicate that ginsenoside rg1 significantly reduced FBG levels and improved the antioxidant and anti-inflammatory capacity of the organism. Therefore, we hypothesize that ginsenoside rg1 achieves its hypoglycemic effect on T2DM animals through an anti-inflammatory and antioxidant mechanism. We further performed subgroup analyses of the primary outcomes, including FBG, superoxide dismutase (SOD), malondialdehyde (MDA), and tumor necrosis factor-α (TNF-α) and interleukin-6 (IL-6) levels. Findings of the subgroup analysis indicate that animal species, ginsenoside rg1 dose, treatment duration, and STZ dose may not be a source of study heterogeneity. Consequently, we hypothesize that heterogeneity could be caused by other differences in the research, for instance, in the design of the study protocol, criteria for successful modeling, characteristics of the samples, and the size of the experimental samples. Therefore, more studies are necessary to provide more precise evidence.

### 4.2 Ginseng and the anti-inflammatory and oxidative effects of ginsenosides

**FIGURE 8:**
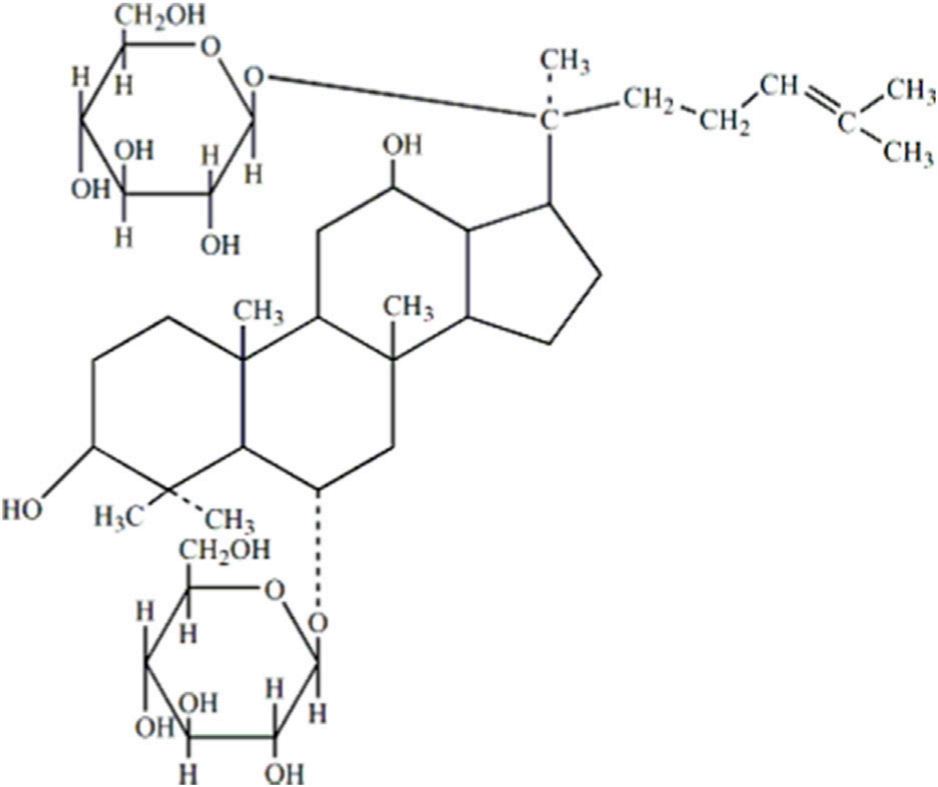
Chemical structure of ginsenoside Rg1.

Chinese herbal medicines (CHM) and their bioactive components usually act on multiple targets and exhibit a pleiotropic spectrum of action. Hence, they may simultaneously affect the underlying processes of diabetes pathogenesis and achieve better efficacy in treating diabetes. Ginsenoside Rg1 (molecular formula: C42H72O14) is primarily obtained from the roots or stems of ginseng, which is derived from the hydride of dammarane, and its chemical structure is shown in Figure 8 (He et al., 2020). Ginsenosides are classified into three categories according to their chemical structures: protopanaxadiol, protriol, and ginsenoside Ro (Song et al., 2017; Kim et al., 2018). According to their different hydroxylation positions because of the core triterpene saponin structure, ginsenosides can be classified into 20(S)-protanediol (PPD) and 20(S)-protatriol (PPT). Different from PPD-type ginsenosides, which are slowly excreted into the bile, PPT-type ginsenoside rg1 is mainly eliminated through rapid hepatobiliary excretion (Gao et al., 2017). Therefore, the molecule-specific structure of Rg1 is a primary parameter in determining the plasma pharmacokinetics of Rg1 and possibly a factor in the drug interaction between Rg1 and its intended molecule. Owing to its distinctive chemical structure, ginsenoside rg1 has a wealth of pharmacological characteristics. Studies have shown that Rg1 can affect a variety of systems in the body, displaying various pharmacological activities (Ahmed et al., 2016; Zhou et al., 2019; Nakhjavani et al., 2020). Ginsenoside rg1 was effective in reducing OS in diabetic rats by participating in the AMPK/Nrf2/HO-1 pathway (Qin et al., 2019). Meanwhile, Rg1 treats T2DM by reducing insulin resistance due to dietary reasons and decreasing the inflammatory response (Fan et al., 2019; Alolga et al., 2020). Current reviews have shown that ginsenosides can be used to treat diabetes through a variety of mechanisms, and ginsenoside rg1 shows the best therapeutic promise as a potential adjuvant for type 2 diabetes (Bai et al., 2018). In this paper, the therapeutic potential of ginsenoside rg1 for diabetes mellitus is elaborated in terms of antioxidant and anti-inflammatory mechanisms.

### 4.3 Oxidative stress is an important mechanism in the pathogenesis of type 2 diabetes, and ginsenosides can be antioxidants

A metabolic malfunction known as OS is caused by an excessive rise in reactive oxygen species (ROS) and an unbalanced antioxidant defense mechanism in the body. Because mitochondria are a major source of ROS, the tight connection between the two organelles via mitochondria-associated membranes (MAMs) means that ROS produced by mitochondria can further contribute to ER stress (Burgos-Moron et al., 2019). OS is recognized as a key risk factor for the onset and progression of diabetes, and the mechanism of occurrence is often multifactorial, covering many cellular signaling pathways (Singh et al., 2022). Under the condition of hyperglycemia, ROS activates many signaling pathways, such as nuclear factor-κβ (NF-κβ) and protein kinase C (PKC), all of which may be related to the dysfunction of the insulin signaling pathway and lead to insulin resistance (Zhang et al., 2020).

OS is the primary factor of β-cell dysfunction and death caused by glucose toxicity and insulin resistance in T2DM (Dinić et al., 2022). Therefore, preserving and restoring functional pancreatic beta cells is a daunting challenge in treating diabetes, regardless of its type (Galicia-Garcia et al., 2020; Batista et al., 2021). In contrast, ginsenoside Rg1, which has free-radical scavenging properties, may play a positive role in surpassing the effects of weakened islet function. Ginseng Rg1 has been demonstrated to reduce D-galactose-induced oxidative damage in the ovary by enhancing T-SOD and GSH-Px activity, decreasing MDA levels, removing free radicals, and activating antioxidant enzymes (He et al., 2017). The growing body of research reveals that ginseng Rg1 has considerable antioxidant potential. Ginseng Rg1 significantly inhibited apoptosis and cystatin-3 activation and reduced ROS and MDA production (Li Q. et al., 2017; Ning et al., 2018). It inhibits NF-κB and inflammatory factor expression, further triggering PI3K/AKT activation and FOXO3 phosphorylation to inhibit apoptosis and reduce diabetes-induced inflammation and OS (Yang et al., 2012; Liu et al., 2021). The liver’s ability to absorb glucose can be further increased, and hyperglycemia can be decreased by activating the PI3K/AKT signaling pathway.

MDA levels may be a key sign of membrane lipid peroxidation, according to the cytotoxic effects of MDA, a byproduct of lipid oxidation. By identifying free radicals, the antioxidant enzyme SOD slows down the aging process (Wang et al., 2018). Therefore, SOD activity and MDA levels are important indicators for assessing antioxidant capacity and responding to the degree of oxidation in the body. The results of this meta-analysis showed that ginsenoside Rg1 could significantly reduce MDA levels and restore SOD activity to exert its antioxidant activity and exert potential therapeutic effects on type 2 diabetes through the antioxidant pathway.

### 4.4 The inflammatory response is an important mechanism in the pathogenesis of type 2 diabetes, and ginsenosides can be anti-inflammatory

Chronic inflammation has been linked to several disorders, including diabetes and cancer (Kim et al., 2017). Scientific evidence suggests that mild chronic inflammation resulting from obesity is the core underlying disease associated with obesity-associated insulin resistance and T2DM (Lontchi-Yimagou et al., 2013). Several preclinical and clinical investigations have reported a clear causal link between mild aseptic inflammation and metabolic illnesses, such as T2DM (Akash et al., 2012; Böni-Schnetzler and Meier, 2019). Additionally, several preclinical and clinical investigations have revealed a causal link between mild aseptic inflammation and metabolic illnesses, such as T2DM. The beta-cell function of the pancreas deteriorates under hypoxic stress, leading to decreased insulin secretion and, eventually, hyperglycemia. In contrast to acute inflammation, in the chronic inflammation of metabolic diseases, pro-inflammatory cytokines and chemokines expand and infiltrate throughout the organ system. Therefore, in islets, elevated innate immune cells and pro-inflammatory mediators cause a decrease in beta cell quality and function (Donath and Halban, 2004; Donath et al., 2013). In obesity, adipose tissue macrophages transform and secrete a variety of pro-inflammatory cytokines that can impair insulin signaling and thus promote the progression of insulin resistance (Zatterale et al., 2020).

Research has shown that ginsenoside Rg1 may control the activity of inflammatory signaling pathways, such as nuclear factor-kB and activator protein-1, and prevent the generation of pro-inflammatory cytokines. The specific mechanisms include the prevention of amyloid β accumulation and microglia activation by inhibiting NF-кB, phospholipase C-γ1 pathway, and downregulation of toll-like receptor 3 and 4 expression, thereby reducing basal inflammation (Hu et al., 2011; Zhao et al., 2014; Li Y. et al., 2017). Animal models have also shown that ginsenoside Rg1 has anti-inflammatory properties. These findings indicate that ginsenoside Rg1 plays a significant role in macrophage-mediated inflammatory responses by regulating the NF-kB or Akt/mTOR signaling pathways through various mechanisms of action (Kim et al., 2017). Ginsenoside Rg1 suppresses IL-6 mRNA and protein expression by suppressing the activation of the NF-kB signaling pathway (Gao et al., 2015; Lee et al., 2015).

Current studies have shown that ginsenoside rg1 can treat various diseases by inhibiting excessive inflammatory pathways, preventing apoptosis, and modulating the immune system. Ginsenoside Rg1 can protect the liver by, among other things, blocking the toll-like receptor 4 signaling pathway, inhibiting the NF-кB signaling pathway, activating AMPK, activating the inflammasome and ER stress, and raising Nrf2 production and translocation (Alana et al., 2012). Meanwhile, the glucocorticoid-like anti-inflammatory effects and immunomodulatory, antioxidant, and anti-apoptotic properties of ginsenoside Rg1 make it a potential therapeutic agent for the treatment of sepsis (Song et al., 2013; Zou et al., 2013; Juan et al., 2015; Su et al., 2015). Because diabetes mellitus is closely associated with chronic inflammation, it is worth investigating and focusing on whether ginsenoside Rg1 may prevent β-cell death or encourage its regeneration for the treatment of diabetes mellitus by lowering inflammation.

### 4.5 Other hypoglycemic mechanisms of ginsenoside Rg1 that alleviate diabetic complications

Furthermore, as ginsenoside Rg1 has low oral bioavailability, it must be deglycosylated and transformed into secondary saponins before it can be absorbed and used in circulation. Existing research has indicated that the majority of ginsenoside deglycosylation occurs in the gastrointestinal tract in response to the activity of intestinal microbes (Peng et al., 2022). As a result, research has demonstrated that ginsenoside Rg1 may be utilized as a dietary supplement alongside prebiotics to treat type 2 diabetes by controlling gut flora (Peng et al., 2022). Because the original search of the literature revealed that the current randomized controlled trials on the treatment of type 2 diabetes by rg1 through the regulation of intestinal flora were insufficient for meta-analysis, we abandoned the study of this mechanism.

Various experimental data have shown that ginsenoside Rg1 is effective in not only lowering BG in type 2 diabetic animals but also alleviating diabetic complications. Ginsenoside Rg1 reduces NF-κB expression and inflammatory vesicle production and attenuates OS and apoptosis in myocardial tissue, thereby alleviating cardiac insufficiency in type 2 diabetic mice (Yu et al., 2016). In studies in diabetic animal models, ginsenoside Rg1 can effectively alleviate the effects of aldosterone-induced OS and reduce the metabolites of ROS to prevent membranous nephropathy in rats while improving the inflammatory response and pathological changes of the kidney through various anti-inflammatory mechanisms (Liu et al., 2021). By establishing an animal model of obesity induced by high-fat and high-sugar diets in mice, ginsenoside Rg1 could induce AMPK activation, inhibit adipogenesis, reduce lipid deposition of fat, play a role in protecting the liver in an anti-obesity manner, and effectively reduce aspartate aminotransferase and alanine aminotransferase indexes (Tian et al., 2017). Ginsenoside Rg1 has also been studied and reported for the prevention of DR. In db/db diabetic retinopathy mouse models, ginsenoside Rg1 intervention can activate the IRS-1/Akt/GSK3β signaling pathway in the early stages of DR, block tau protein-induced neurodegeneration at retinal ganglion cell synapses, and improve visual function (Gao et al., 2020). Meanwhile, ginsenoside Rg1 can improve the angiogenesis of endothelial cells and promote wound closure in diabetic foot ulcers (Yang et al., 2012).

### 4.6 Summary and limitations to the study

In this review, we conducted a meta-analysis of preclinical trials regarding ginsenoside rg1 for the treatment of T2DM. The objective was to evaluate the hypoglycemic effect and the antioxidant and anti-inflammatory properties of ginsenoside rg1 in the treatment of type 2 diabetes. Studies have shown that people with type 2 diabetes are at a significantly increased risk of developing complications of type 2 diabetes when chronic hyperglycemia is not effectively controlled. For people with diabetes who have uncontrolled hyperglycemia, the likelihood of developing Alzheimer’s disease later in life is greatly increased (Nowaczewska et al., 2019). Therefore, aggressive treatment of hyperglycemia becomes a key measure in the treatment of diabetes. The findings showed that ginsenoside rg1 was apparently correlated with antioxidant factors and pro-inflammatory cytokines. It inhibited the generation of MDA, TNF-α, and IL-6 and promoted SOD production. Current evidence shows that ginsenoside rg1 can effectively control hyperglycemia due to type 2 diabetes through antioxidant and anti-inflammatory mechanisms. This meta-analysis is based on animal experiments; therefore, it cannot represent the results of clinical trials. However, it still has a certain reference value and guiding significance for future experiments.

However, there are some unavoidable limitations in this study. First, in terms of study quality, none of the studies described the methods used to conceal allocation order, blinded interventions, or randomized outcome assessments. Furthermore, some studies did not provide detailed baseline characteristics. At the same time, given the inadequate methodological quality of some studies, the results of this study should be construed with care, and more qualitative studies are necessary in the future. Second, the high heterogeneity of the results diminished their dependability. Although we attempted to explore potential sources of heterogeneity using subgroup analysis, it seems unproductive. Different study protocols and intervention procedures in animal experiments may be a potential cause of the high heterogeneity. Third, there is minimal evidence for the efficacy of ginsenoside Rg1 in the treatment of type 2 diabetes *in vitro* and *in vivo* trials. Therefore, clinical investigations are required. However, the transition from preclinical to clinical investigations has been delayed by concerns with bioavailability. Fourth, based on the studies we included, investigators have set the dose and duration of Rg1 therapy in various ways. Consequently, current reports make it difficult to obtain reliable, effective doses and appropriate treatment durations. More research in this area should address this issue. Finally, we did not conduct further meta-analyses of relevant indicators because data for certain indicators only existed in separate studies. These indicators require more attention in the future.

## 5 Conclusion

In this meta-analysis, the findings showed that ginsenoside rg1 was apparently correlated with antioxidant factors and pro-inflammatory cytokines. It inhibited the generation of MDA, TNF-α, and IL-6 and promoted SOD production. The findings of the present study can fully demonstrate the antioxidant and anti-inflammatory properties of ginsenoside rg1. However, the low methodological quality of the included studies and publication bias may weaken the validity of the findings. The hypoglycemic effects of ginsenosides in type 2 diabetes require more rigorous experimental design and more comprehensive studies.

## Data Availability

The original contributions presented in the study are included in the article/Supplementary Material. Further inquiries can be directed to the corresponding author.
