# Supplementary material for: Antioxidant and anti-inflammatory properties of ginsenoside Rg1 for hyperglycemia in type 2 diabetes mellitus: systematic reviews and meta-analyses of animal studies
Source: Front Pharmacol. 2023 Sep 8;14:1179705. doi: 10.3389/fphar.2023.1179705 (PMC10514510; doi:10.3389/fphar.2023.1179705)
Supplement: Supplementary file 2 [file Table1.DOCX]

TABLE| S2 summary table describing the Ginsenoside Rg1.

| **Study（years）** | **Source** | **Purity (%)** | **Quality control reported?** |
| --- | --- | --- | --- |
| Feng Jie, 2010 | The National Institute for the Control of Pharmaceutical and Biological Products,China | 98% | Y- HPLC |
| Zhen et al., 2016 | Kunming Medical University,china | 95%， | Y- HPLC |
| Yao Jianchao, 2016 | NR | NR | NR |
| Ruan et al., 2021 | NR | NR | NR |
| Yang et al., 2020 | Sleike Jingda Co., LTD,Shanghai,China | NR | NR |
| Li Xin, 2015 | Chengguang Biotechnology Co. LTD,Baoji,China） | NR | NR |
| Wu et al., 2021 | Lingnan Pharmaceutical Co,[Korea](C:/Users/ASUS/AppData/Local/youdao/dict/Application/9.1.2.0/resultui/html/index.html#/javascript:;) | ≥98% | Y- HPLC |
| Li et al., 2022 | Durst Biotechnology Co., LTD,Chengdu,China | ≥98% | Y- HPLC |
| Chen et al., 2020 | Kunming Medical University,China | 95% | Y- HPLC |
| Ma et al., 2022 | Solarbio (China) | NR | NR |
| Yang et al., 2012 | The National Institute for the Control of Pharmaceutical and  Biological Products,China | ＞96% | Y- HPLC |
| Gao et al., 2019 | the Winherb Medical Technology Company (Shanghai, China). | 98% | Y- HPLC |
| Yuan et al., 2019 | Sigma-Aldrich, St. Louis, MO, USA | NR | NR |
| Liu et al., 2021 | Sigma-Aldrich, St. Louis, MO, USA | ≥90% | Y- HPLC |
| Yaoyao Yin and Junxia Wang,2017 | Nanjing plant industry co., LTD,China | NR | NR |
| Tian et al., 2017 | Kunming Medical University,China | >98% | Y- HPLC |
| Yu et al., 2015 | Jilin University,Jilin,China | >98% | Y- HPLC |
| Peng et al., 2022 | Medical University,Kunming,China | 98% | Y- HPLC |

**Abbreviations:** (HPLC: high performance liquid chromatography; NR: no report).
