# Supplementary material for: Antioxidant and anti-inflammatory properties of ginsenoside Rg1 for hyperglycemia in type 2 diabetes mellitus: systematic reviews and meta-analyses of animal studies
Source: Front Pharmacol. 2023 Sep 8;14:1179705. doi: 10.3389/fphar.2023.1179705 (PMC10514510; doi:10.3389/fphar.2023.1179705)
Supplement: Supplementary file 3 [file Table2.DOCX]

TABLES3| subgroup analysis

| **outcome** | **subgroup** | | **No. studies** | **SMD [95%CI]** | ***P* value** | **I^2^(%)** | ***P*-heterogeneity** |
| --- | --- | --- | --- | --- | --- | --- | --- |
| BG | species | rats | ８ | -4.05[-5.41,-2.69] | ＜0.001 | 83 | ＜0.001 |
|  |  | mice | ４ | -2.40[-3.57,-1.22] | ＜0.001 | 71 | 0.01 |
|  | STZ (mg/kg) | <50 | ５ | -4.37[-6.18,-2.56] | ＜0.001 | 81 | ＜0.001 |
|  |  | ≥50 | ４ | -2.56[-4.02,-1.28] | ＜0.001 | 79 | 0.003 |
|  | dosage(mg/kg) | <40 | ５ | -4.40[-6.41,-2.39] | ＜0.001 | 88 | ＜0.001 |
|  |  | ≥40 | ６ | -2.93[-4.17,-1.69] | ＜0.001 | 80 | ＜0.001 |
|  | Duration（week） | <8 | ４ | -2.39[-3.24,-1.54] | ＜0.001 | 50 | 0.11 |
|  |  | ≥8 | ７ | -4.59[-6.36,-2.82] | ＜0.001 | 88 | ＜0.001 |
| TNF-α | species | rats | ７ | -5.64[-7.08,-4.19] | ＜0.001 | 67 | 0.006 |
|  |  | mice | １ | -17.57[-23.71,-11.43] | ＜0.001 | — | — |
|  | STZ (mg/kg) | <50 | ４ | -5.66[-6.77,-4.55] | ＜0.001 | 0 | 0.83 |
|  |  | ≥50 | ３ | -10.21[-18.62,-1.80] | 0.02 | 94 | ＜0.001 |
|  | dosage(mg/kg) | <40 | ３ | -5.35[-6.48,-4.21] | ＜0.001 | 0 | 0.98 |
|  |  | ≥40 | ４ | -6.27[-9.25,-3.29] | ＜0.001 | 83 | ＜0.001 |
|  | Duration（week） | <8 | １ | -5.66[-8.13,-3.20] | ＜0.001 | — | — |
|  |  | ≥8 | ６ | -5.68[-7.35,-4.02] | ＜0.001 | 72 | 0.003 |
| IL-6 | species | rats | ６ | -5.63[-7.06,-4.19] | ＜0.001 | 61 | 0.02 |
|  |  | mice | １ | -12.19[-16.50,-7.89] | ＜0.001 | — | — |
|  | STZ (mg/kg) | <50 | ３ | -6.09[-8.65,-3.52] | ＜0.001 | 71 | 0.03 |
|  |  | ≥50 | ３ | -6.50[-10.10,-2.89] | ＜0.001 | 85 | 0.002 |
|  | dosage(mg/kg) | <40 | ３ | -6.40[-7.73,-5.07] | ＜0.001 | 0 | 0.41 |
|  |  | ≥40 | ３ | -4.86[-7.03,-2.69] | ＜0.001 | 69 | 0.04 |
|  | Duration（week） | <8 | １ | -3.91[-5.75,-2.06] | ＜0.001 | — | — |
|  |  | ≥8 | ５ | -6.04[-7.65,-4.42] | ＜0.001 | 60 | 0.04 |
| ＳＯＤ | species | rats | ６ | 3.88[2.32,5.43] | ＜0.001 | 84 | ＜0.001 |
|  |  | mice | １ | 2.42[1.21,3.63] | ＜0.001 | — | — |
|  | STZ (mg/kg) | <50 | ３ | 2.56[1.06,4.05] | ＜0.001 | 73 | 0.02 |
|  |  | ≥50 | ２ | 5.79[4.52,7.06] | ＜0.001 | 0 | 0.83 |
|  | dosage(mg/kg) | <40 | ４ | 3.98[1.87,6.09] | ＜0.001 | 89 | ＜0.001 |
|  |  | ≥40 | ３ | 3.07[1.50,4.64] | ＜0.001 | 67 | 0.05 |
|  | Duration（week） | <8 | ２ | 2.33[1.44,3.22] | ＜0.001 | 0 | 0.84 |
|  |  | ≥8 | ５ | 4.25[2.37,6.12] | ＜0.001 | 86 | ＜0.001 |
| MDA | STZ (mg/kg) | <50 | ３ | -3.40[-5.36,-1.44] | ＜0.001 | 78 | 0.01 |
|  |  | ≥50 | ２ | -3.65[-4.83,-2.47] | ＜0.001 | 37 | 0.21 |
|  | dosage(mg/kg) | <40 | ４ | -3.95[-5.65,-2.25] | ＜0.001 | 83 | ＜0.001 |
|  |  | ≥40 | ２ | -3.36[-4.55,-2.18] | ＜0.001 | 0 | 0.38 |
|  | Duration（week） | <8 | １ | -4.02[-5.90,-2.14] | ＜0.001 | — | — |
|  |  | ≥8 | ５ | -3.72[-5.07,-2.38] | ＜0.001 | 77 | 0.002 |
