# Supplementary material for: Antioxidant and anti-inflammatory properties of ginsenoside Rg1 for hyperglycemia in type 2 diabetes mellitus: systematic reviews and meta-analyses of animal studies
Source: Front Pharmacol. 2023 Sep 8;14:1179705. doi: 10.3389/fphar.2023.1179705 (PMC10514510; doi:10.3389/fphar.2023.1179705)
Supplement: Supplementary file 4 [file DataSheet1.docx]

Figure 1| The egger test of BG


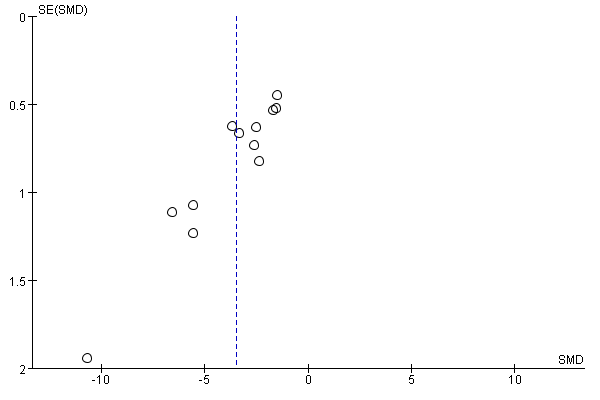


Figure 2 | the funnel plots of BG


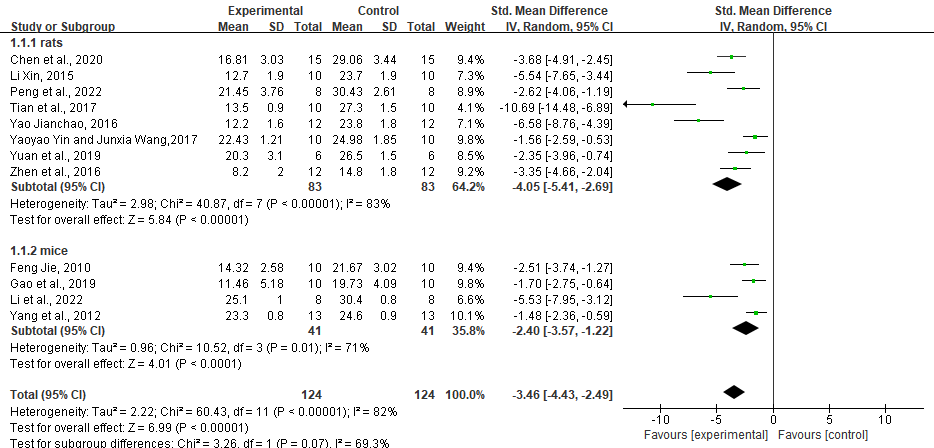


Figure 3 | Subgroup analysis of blood glucose with respect to species


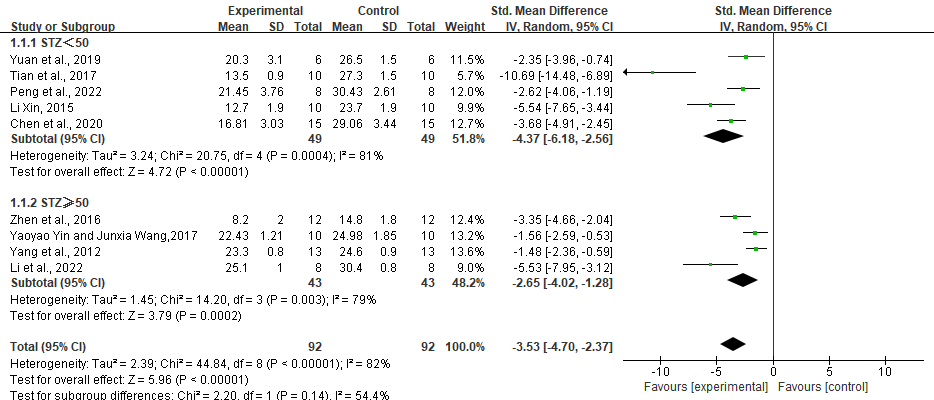


Figure 4 | Subgroup analysis of blood glucose with respect to STZ


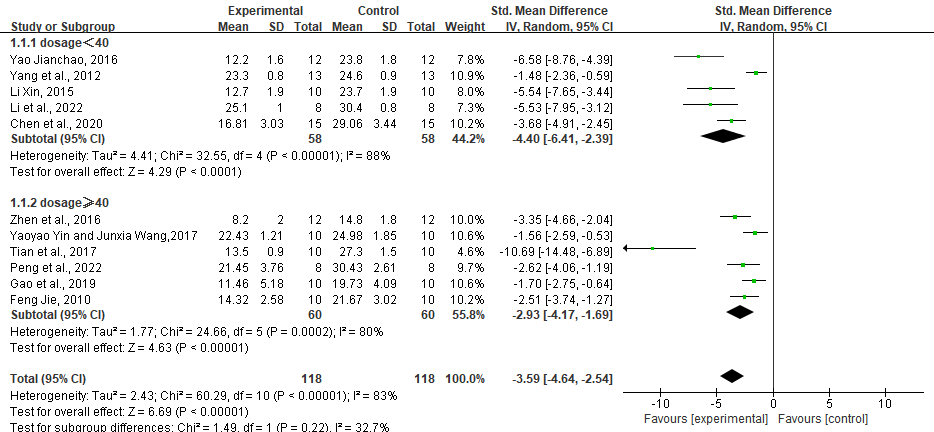


Figure 5 | Subgroup analysis of blood glucose with respect to dosage.


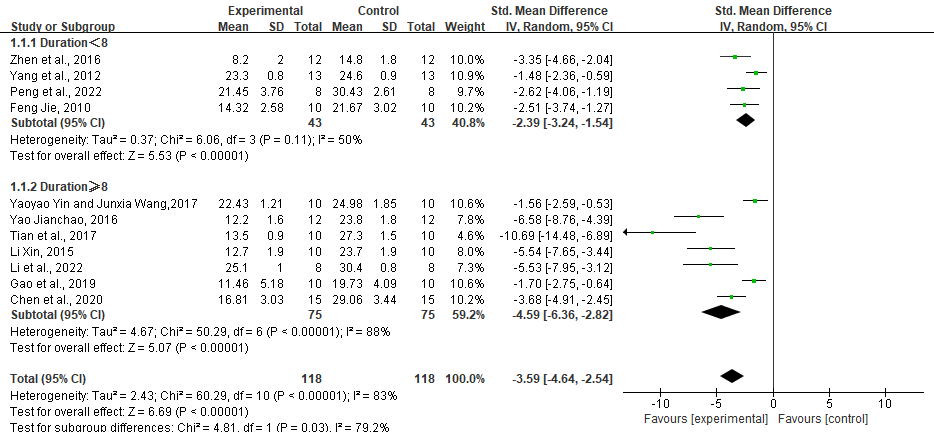


Figure 6 | Subgroup analysis of blood glucose with respect to duration.


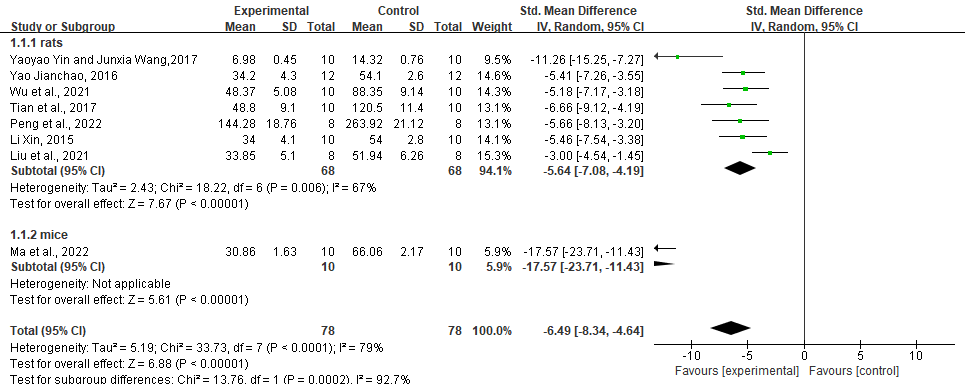


Figure7 | Subgroup analysis of TNF-α with respect to species.


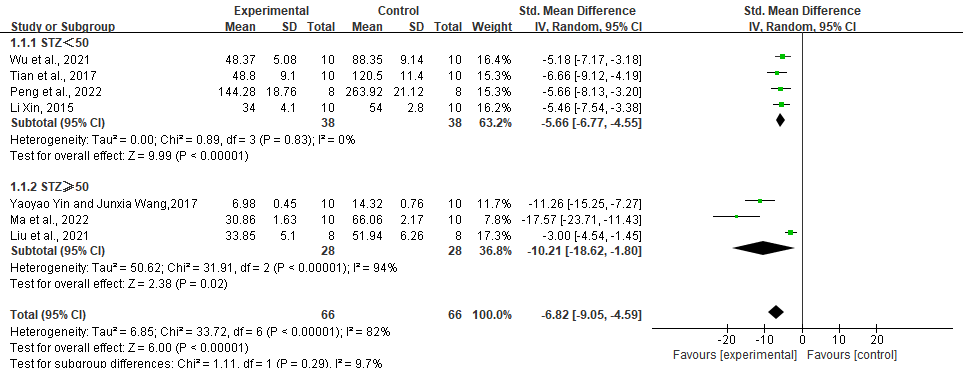


Figure 8 | Subgroup analysis of TNF-α with respect to STZ.


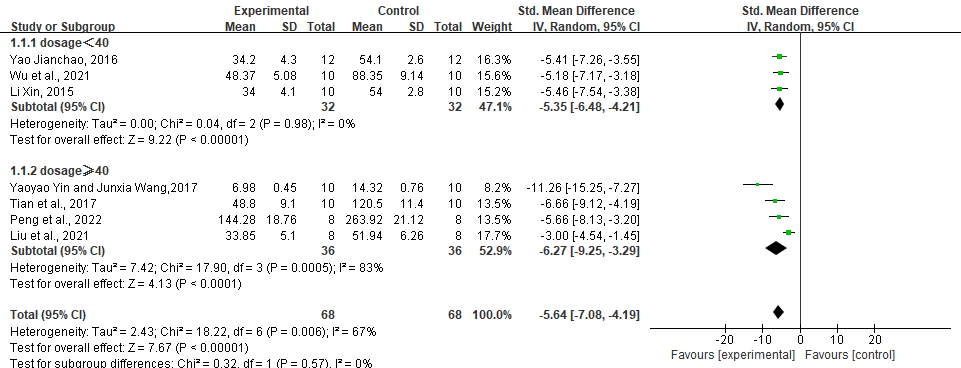


Figure 9 | Subgroup analysis of TNF-α with respect to dosage.


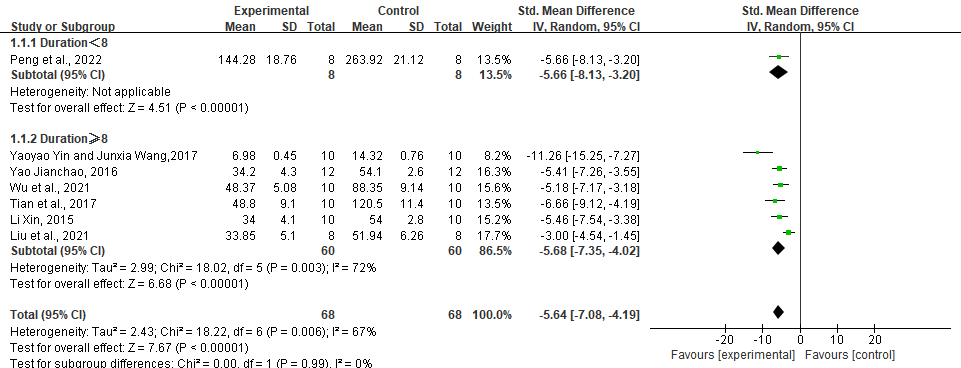


Figure 10 |Subgroup analysis of TNF-α with respect to duration.


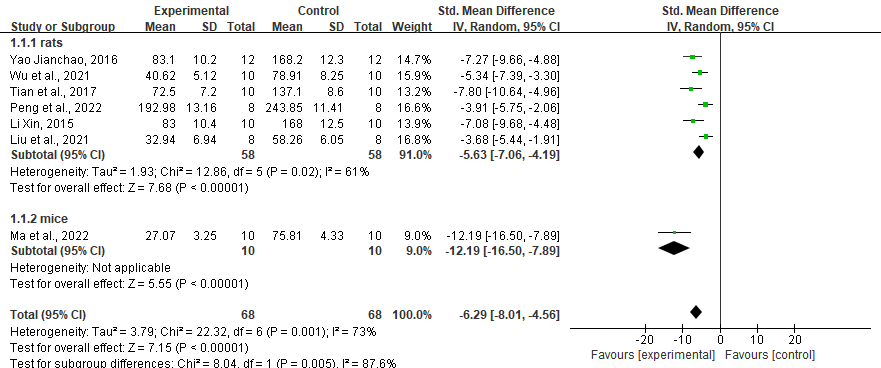


Figure 11 | Subgroup analysis of IL-6 with respect to species.


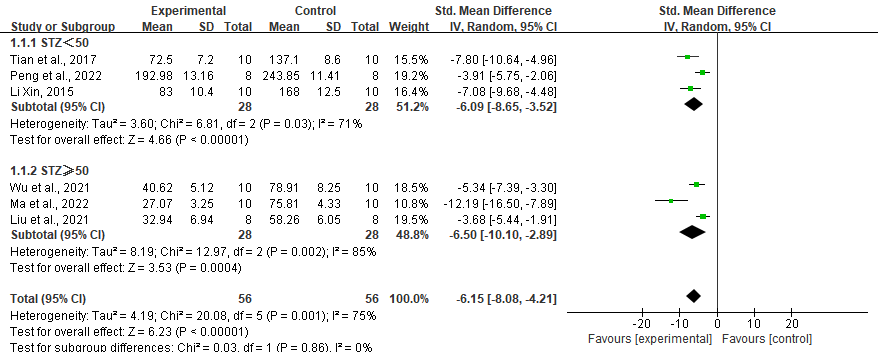


Figure 12 | Subgroup analysis of IL-6 with respect to STZ.


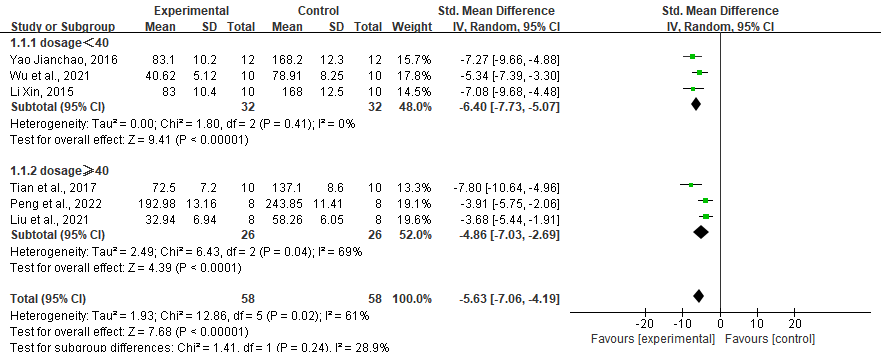


Figure 13| Subgroup analysis of IL-6 with respect to dosage.


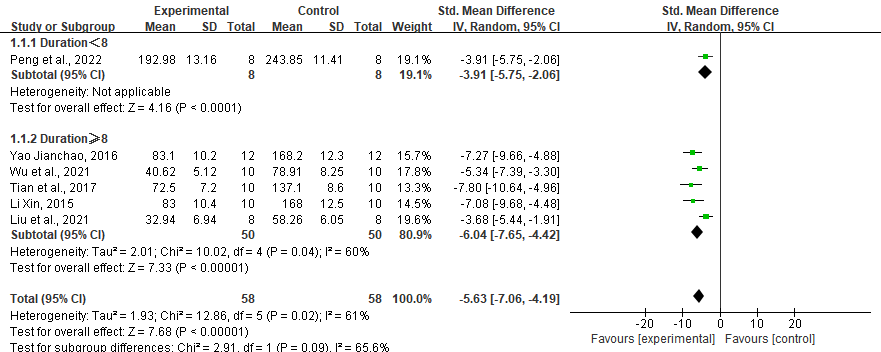


Figure 14 | Subgroup analysis of IL-6 with respect to duration.


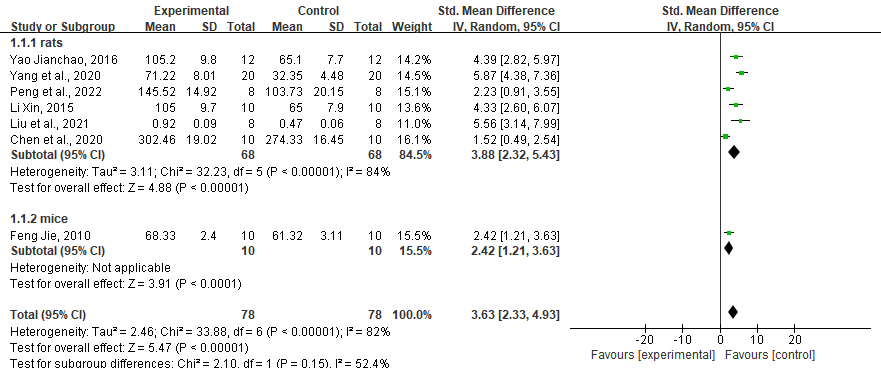


Figure 15| Subgroup analysis of SOD with respect to species.


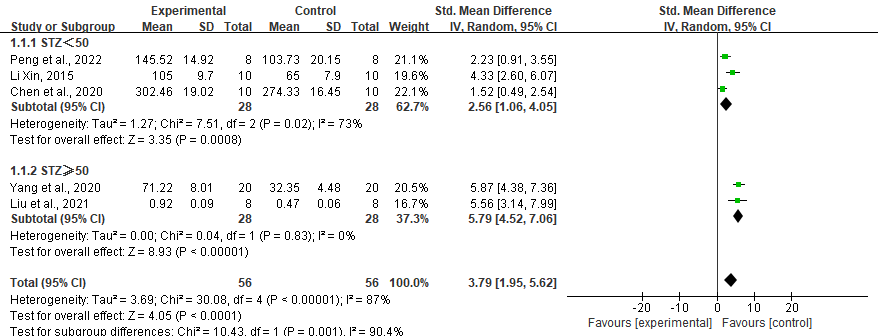


Figure 16| Subgroup analysis of SOD with respect to STZ.


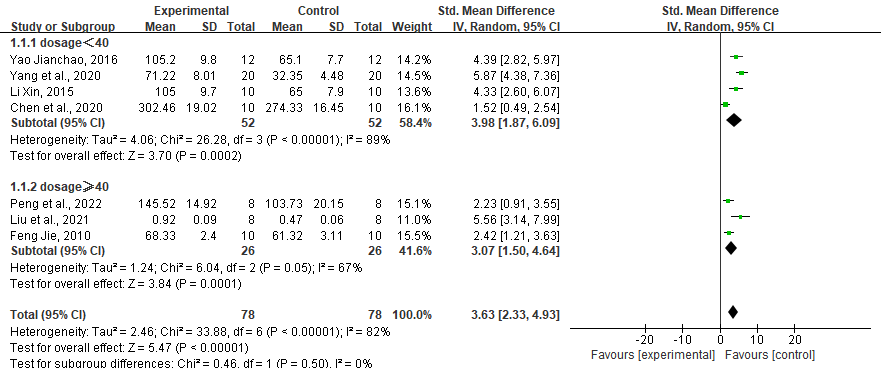


Figure 17| Subgroup analysis of SOD with respect to dosage.


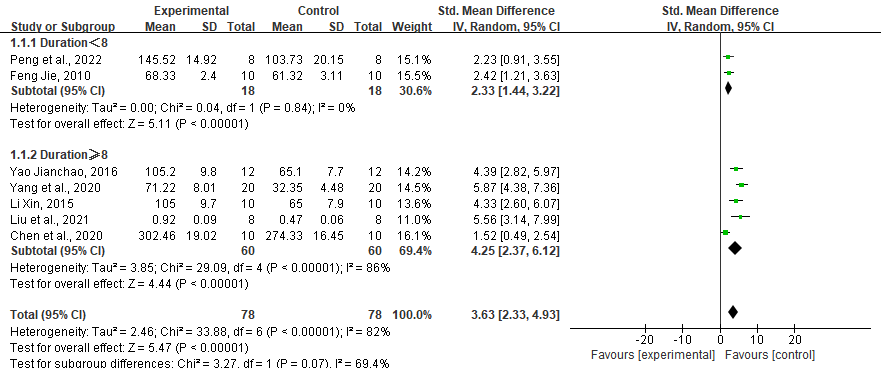


Figure 18| Subgroup analysis of SOD with respect to duration.


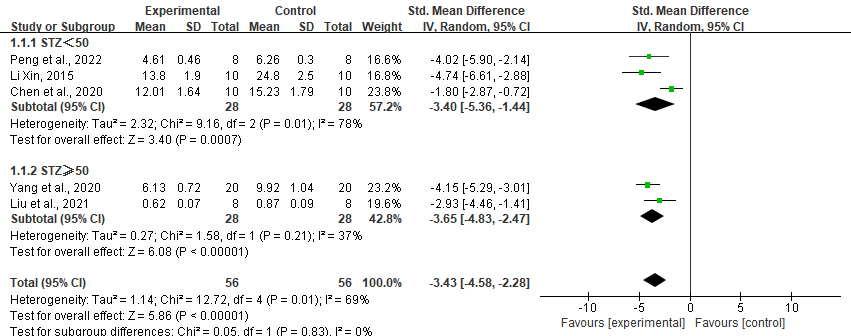


Figure 19| Subgroup analysis of MDA with respect to STZ.


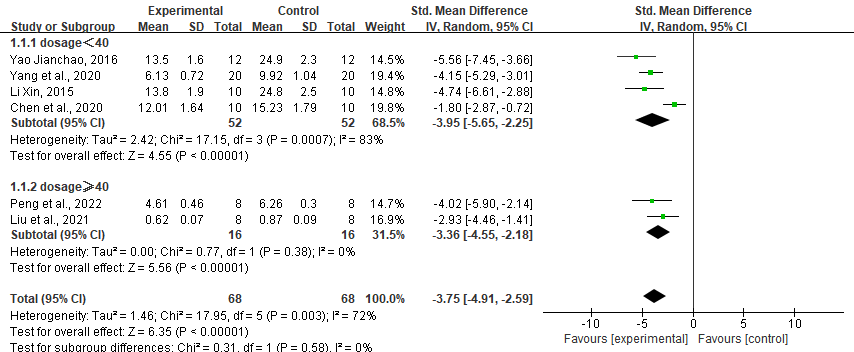


Figure 20| Subgroup analysis of MDA with respect to dosage.


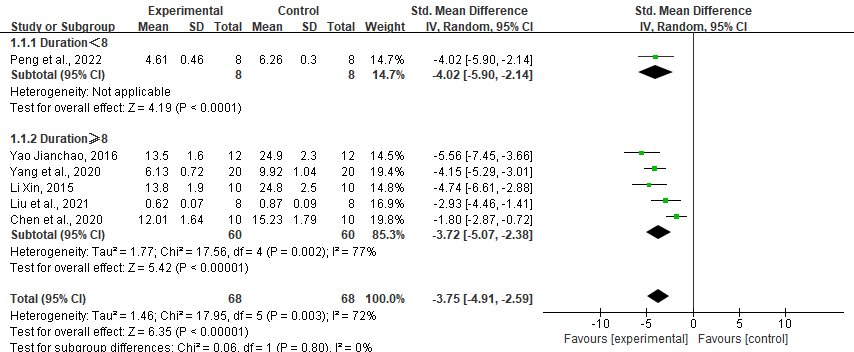


Figure 21| Subgroup analysis of MDA with respect to duration.
